# Nuclear localisation of LASP-1 correlates with poor long-term survival in female breast cancer

**DOI:** 10.1038/sj.bjc.6605685

**Published:** 2010-05-11

**Authors:** J J Frietsch, T G P Grunewald, S Jasper, U Kammerer, S Herterich, M Kapp, A Honig, E Butt

**Affiliations:** 1 Institute for Clinical Biochemistry and Pathobiochemistry, University of Wuerzburg, Grombuehlstr. 12, 97080 Wuerzburg, Germany; 2 Department of Pediatrics, Children's Cancer Research Center (CCRC), Laboratory of Functional Genomics and Transplantation Biology, Klinikum rechts der Isar, Technische Universität München, Kölner Platz 1, 80804 Munich, Germany; 3 Department of Obstetrics and Gynecology, University of Wuerzburg, Josef-Schneider Str 4, 97080 Wuerzburg, Germany

**Keywords:** LASP-1, Ki67, PDEF, p53, breast cancer

## Abstract

**Background:**

LIM and SH3 protein 1 (LASP-1) is a nucleo-cytoplasmatic signalling protein involved in cell proliferation and migration and is upregulated in breast cancer *in vitro* studies have shown that LASP-1 might be regulated by prostate-derived ETS factor (PDEF), p53 and/or *LASP1* gene amplification. This current study analysed the prognostic significance of LASP-1 on overall survival (OS) in 177 breast cancer patients and addressed the suggested mechanisms of LASP-1-regulation.

**Methods:**

Nucleo-cytoplasmatic LASP-1-positivity of breast carcinoma samples was correlated with long-term survival, clinicopathological parameters, Ki67-positivity and PDEF expression. Rate of *LASP1* amplification was determined in micro-dissected primary breast cancer cells using quantitative RT–PCR. Cell-phase dependency of nuclear LASP-1-localisation was studied in synchronised cells. In addition, LASP-1, PDEF and p53 expression was compared in cell lines of different tumour entities to define principles for LASP-1-regulation.

**Results:**

We showed that LASP-1 overexpression is not due to *LASP1* gene amplification. Moreover, no correlation between p53-mutations or PDEF-expression and LASP-1-status was observed. However, nuclear LASP-1-localisation in breast carcinomas is increased during proliferation with peak in G2/M-phase and correlated significantly with Ki67-positivity and poor OS.

**Conclusion:**

Our results provide evidence that nuclear LASP-1-positivity may serve as a negative prognostic indicator for long-term survival of breast cancer patients.

Breast cancer is expected to account for 26% of all new cancer cases among women in the western world of which 89% will survive 5 years after diagnosis (Jemal *et al*, 2007). In spite of the significant improvements in diagnostic and therapeutic modalities for the treatment of cancer patients, metastasis still composes the major cause of mortality being responsible for 60% of breast cancer deaths (Hanahan and Weinberg, 2000). Metastatic disease remains generally incurable with a median survival time of only a few years. Regardless of the increase in its incidence, mortality related to breast cancer is decreasing because of raised awareness and screening, as well as multidisciplinary treatment. The introduction of endocrine therapy and the treatment with trastuzumab (Herceptin) in patients with HER-2/neu overexpression reduced the rates of recurrence by 50% and significantly improved survival (Widakowich *et al*, 2007). Nevertheless, breast cancer remains a multi-step process linked to more than one single molecular alteration. Therefore, elucidating genes that are overexpressed in breast cancer cells may yield promising targets for novel therapeutic agents (Mauriac *et al*, 2005).

The LIM and SH3 domain protein (LASP-1) was initially identified from a cDNA library of breast cancer metastases. It became the first member of a newly defined LIM-protein subfamily of the nebulin group characterised by the combined presence of LIM and SH3 domains (Grunewald and Butt, 2008). LASP-1 is localised within multiple sites of dynamic F-actin assembly such as focal contacts, lamellipodia and membrane ruffles it binds to the specific shuttle proteins Zyxin and lipoma preferred partner (LPP) and is involved in cell migration and proliferation (Schreiber *et al*, 1998; Chew *et al*, 2002; Butt *et al*, 2003; Nakagawa *et al*, 2006). Silencing of LASP-1 by RNA-interference in various cancer cell lines resulted in strong inhibition of proliferation and migration with cell cycle arrest in G2/M-phase (Grunewald *et al*, 2006, 2007b).

LIM and SH3 protein 1 mRNA is expressed ubiquitously at low basal levels in all normal human tissues, but is overexpressed in metastatic human breast cancer (Grunewald and Butt, 2008) and ovarian cancer (Grunewald *et al*, 2007b; Dimova *et al*, 2009). In a recent case–control study, LASP-1-expression correlated significantly with tumour size and nodal-positivity (Grunewald *et al*, 2007a). Albeit the protein is predominantly situated at focal adhesions, nuclear localisation of the protein could be clearly detected by confocal microscopy and western blots of cytosolic and nuclear preparations from various breast cancer cell lines (Grunewald *et al*, 2007a). These data prompted us to further investigate the long-term survival in relation to nuclear and cytosolic LASP-1-localisation in a large well-characterised cohort of breast cancer patients and to analyse the nuclear LASP-1-localisation in the different phases of the cell cycle.

In invasive breast cancer cells, LASP-1-expression was significantly inversely affected by prostate-derived ETS factor (PDEF), a transcription factor known to repress a variety of genes that are possibly involved in oncogenesis, such as the apoptosis regulator survivin (Ghadersohi *et al*, 2007) and the pro-invasive protease uPA (Turner *et al*, 2008).

Real-time PCR analysis confirmed upregulation of *LASP1* mRNA in PDEF-deficient invasive and highly metastatic breast cancer cells (MBA-MB-231, BT-549), while in the non-invasive MCF-7 breast cancer cell line, endogenously expressing PDEF, a reduced LASP-1 protein level was detected (Turner *et al*, 2008).

In a study conduced with hepatocellular carcinoma (HCC), LASP-1 was repressed by wild-type p53 at the transcriptional level (Wang *et al*, 2009). Functional negative p53 mutations led to increased LASP-1-expression and to a more aggressive HCC phenotype (Wang *et al*, 2009).

In this study, we aimed to determine whether the PDEF level or mutations of the tumour suppressor p53 represent a general mechanism of LASP-1 deregulation in human cancer. Thus, we analysed several tumour cell lines of different entities as well as breast cancer tissue for LASP-1-expression in correlation to PDEF protein concentration and p53 status.

As *LASP1* gene amplification was reported earlier in one breast cancer cell line (Tomasetto *et al*, 1995b) and in 40% of crude extracts of lymph nodes derived from metastatic breast cancer (Tomasetto *et al*, 1995a) and was accounted for the principal cause of LASP-1 overexpression, we also re-analysed the rate of *LASP1* gene amplification in individual micro-dissected primary breast cancer cells. It is noteworthy that the *LASP1* gene maps to a region (17q12) that is altered in 20–30% of human breast cancers (Tomasetto *et al*, 1995a, 1995b) and tumours bearing amplifications of 17q11-21 are associated with an adverse prognosis because of increased resistance to chemotherapy and endocrine therapy (Ross and Fletcher, 1999).

## Materials and methods

### Tissue samples

The studies were performed with approval of the ethics committee of the University of Wuerzburg. Paraffin-embedded tissue samples of surgical biopsies from 177 patients with invasive breast cancer were obtained from the Department of Pathology of the University of Wuerzburg. The patients were aged from 32 to 85 (mean 55.3±11.9) years. All carcinomas were mainly collected from patients that underwent wide excisions.

Grading of malignancy of ductal carcinomas was evaluated according to the Scarff, Bloom and Richardson criteria as suggested by Nottingham City Hospital Pathologists (Dalton *et al*, 2000). Tumour staging was performed according to parameters of the TNM system (Singletary and Connolly, 2006).

Ten paraffin-embedded breast tissue samples from reduction mammoplasties were used as control tissue to obtain reference DNA for the evaluation of the quantitative RT–PCR results.

### Immunohistochemistry

For immunohistochemical staining procedures tissue sections were cut from regular paraffin-embedded tissue at 2–3 *μ*m. Sections were placed onto APES (3-amino-propyltriethoxy-silane; Roth, Karlsruhe, Germany) coated slides, dewaxed twice in xylene for 10 min, rehydrated in graded ethanol (two changes in 96%, one change in 70%, one change with distilled water) and in TRIS-buffered saline (25 mM TRIS/HCl, pH 7.4, 137 mM NaCl, 2.7 mM KCl) for 1 min each. For antigen retrieval, sections were subjected to heat pretreatment by boiling in 0.01 M of sodium citrate buffer (pH 6.0) for 5 min in a microwave oven (800 W s^–1^). Endogenous peroxidase was blocked by incubation in 3.0% hydrogen peroxide in methyl alcohol for 5 min, washed in PBS and incubated in Beriglobin (Aventis-Behring GmbH, Marburg, Germany) 1 : 10 in PBS at room temperature (RT) for 15 min to prevent unspecified attachments. Slides were then incubated with the polyclonal anti-LASP-1 antibody (Butt *et al*, 2003) diluted 1 : 1000 in antibody diluent (DAKO, Hamburg, Germany) or with Ki67 antibody (DAKO) diluted 1 : 100 in antibody diluent at 4 °C overnight followed by EnVision/rabbit detection system (DAKO) for 30 min at RT. 3,3′-Diaminobenzidine (DAB; DAKO) was used as chromogen and sections were counterstained in haematoxylin (Mayers, Sigma, Deisenhofen, Germany), dehydrated through graded ethanol (in the inverse way as described above) and embedded in Entelan (Merck, Darmstadt, Germany). The specificity of the LASP-1 antibody is shown in Supplementary Figure 1.

For PDEF staining, slices were incubated in Beriglobin, diluted 1 : 50 in PBS at RT for 15 min before incubation with polyclonal anti-PDEF antibody (Invitrogen, Karlsruhe, Germany) diluted 1 : 50 in ‘antibody diluent’ (DAKO) at 4 °C overnight. After washing with PBS, the slides were incubated for 15 min with biotinylated secondary antibody followed by 15 min incubation with streptavidin-HRP (both LSAB2 system DAKO). The specificity of the PDEF antibody is shown in Supplementary Figure 2.

### Evaluation of immunohistochemical LASP-1 staining and LASP-1-IRS

After staining procedure, the slides were screened and scored as previously described (Grunewald *et al*, 2007a). To assess the role of LASP-1 in human breast cancer, we examined its expression in 177 breast carcinoma samples from patients selected randomly from January 1985 to December 2007. Semi-quantitative evaluation of LASP-1 immunostaining was carried out by defining the percentage of positive cells and the staining intensity as described below. For positive controls, breast cancer sections previously described as highly LASP-1-positive (Grunewald *et al*, 2006, 2007a) were used. No staining was observed in negative controls with omitted primary antibody or with pre-immune serum (data not shown).

Scoring of cytosolic LASP-1-expression was carried out in analogy to the scoring of hormone receptor Immune Reactive Score (IRS) ranging from 0 to 12 according to Remmele *et al* (Remmele and Stegner, 1987), which is used routinely in surgical pathology for the quantification of hormone receptor expression in mammary carcinoma.

**Figure 1:**
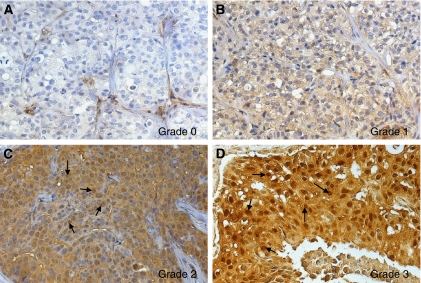
Representative images of heterogeneous LASP-1-expression in human invasive breast cancer. Immunohistochemical staining of LASP-1 (DAB, brown, magnification × 20). (**A**) No LASP-1-expression (grade 0). (**B**) Low LASP-1-expression (grade 1). (**C**) Medium LASP-1-expression (grade 2). (**D**) High LASP-1-expression (grade 3). Arrows in (**C**) and (**D**) point to LASP-1-positive nuclei.

The percentage of LASP-1-postitive stained cells was scored in five grades (grade 0=0–19%, grade 1=20–39%, grade 2=40–59%, grade 3=60–79% and grade 4=80–100% LASP-1-expressing tumour cells) by examining 10 high-power fields ( × 40 magnification) in each tissue sample. In addition, the intensity of LASP-1-expression by the tumour cells was determined (score 0=none, score 1=low, score 2=moderate, score 3=strong). The multiplication of these two grading scores (% LASP-1-positive tumour cells × staining intensity) calculates the immunoreactive score for LASP-1-expression (LASP-1-IRS). Examples for the very heterogeneous LASP-1-expression in invasive breast cancer are given in Figure 1.

For better statistical discrimination, samples scored with cytosolic LASP-1-IRS <5 were classified as LASP-1-negative, those with LASP-1-IRS >5 as LASP-1-positive.

Nuclear LASP-1-staining was scored by determining percentage of positive nuclei regardless of cytosolic LASP-1-expression and cytosolic staining intensity. Samples were considered as nuclear-positive when 10% or more cells showed nuclear LASP-1 staining. Examples for nuclear LASP-1 staining are observed in Figures 1C and D.

The immunomarkers c-erbB2 (HER-2/neu), oestrogen receptor and progesterone receptor assessed in this study had been previously detected by standard immunohistochemistry and were drawn from the archival database of the Department of Pathology of the University of Wuerzburg.

### Scoring of Ki67-positivity

Immunohistochemical scoring was performed by counting 10 randomly selected 40 × high-power fields containing representative sections of tumour and calculated as the percentage of positively stained cells to total cells by counting at least 1000 malignant cells. Ki67 ⩾10% nuclear staining was required for a positive classification (Tan *et al*, 2005).

### Statistical analysis

Associations between nuclear or cytosolic LASP-1-localisation were evaluated by multivariate non-parametric analysis using Fisher's exact (F) and Mann–Whitney (M) test. These tests were conducted using Graph Pad Prism Software for Windows (GraphPad Software, Inc., La Jolla, CA, USA). *P*<0.05 were regarded as statistically significant.

### Tissue preparation, micro-dissection, DNA preparation

In all, 64 formalin-fixed paraffin-embedded breast cancer tissue samples of the patient cohort and 10 control breast tissues were placed onto PEN-membrane coated slides (polyethylene naphthalate; Leica, Wetzlar, Germany) were deparaffinised (two changes of xylene, two changes of 96% ethanol, one change of 70% ethanol, one change with distilled water, 1 min each). After staining with haematoxylin for 90 s and eosin for 60 s, all sections were rinsed 3 × with distilled water. The slides were then air-dried at RT and used for micro-dissection. Incubation and staining times were kept as short as possible to enhance DNA recovery and proteinase K digestion (Godfrey *et al*, 2000; Ehrig *et al*, 2001).

Laser capture micro-dissection was performed using the Laser MicroBeam System (Leica LMD 6000; Leica). Tumour tissue (2.0–2.5 mm^2^) was captured into the lid of a 0.5 ml reaction tube and digested with 30 *μ*l proteinase K digestion buffer (50 mM Tris, pH 8.1; 1 mM ethylenediamine tetraacetic acid; 0.5% Tween 20; 3 mU ml^–1^ proteinase K). Subsequently, the tubes were closed in this inverted position and incubated for 50–60 h at 37 °C. Undigested debris was removed by centrifugation at 14 000 **g** for 5 min, and proteinase K was inactivated by incubation at 95 °C for 10 min. The samples can be stored safely for months at −20 °C (Lehmann and Kreipe, 2001).

### Quantitative RT–PCR

Quantitative analysis of genomic *LASP1* DNA was performed by monitoring the increase in fluorescence of the dye SYBR Green (SYBR Green Supermix, Bio-Rad, Munich, Germany) using the iCycler iQ System (Bio-Rad).

Primers were designed to meet specific criteria by using Primer3 software (http://frodo.wi.mit.edu) (Rozen and Skaletsky, 2000) and were obtained from Operon Biotechnologie GmbH (Cologne, Germany). The sequences of the primers used for *LASP1* DNA amplification were 5′-TGTCTCCTGACTGGTTGCGT-3′, and 5′-TGATCTGGTCCTGGGTCTTC-3′. Primers for GAPDH were used as internal reference gene: 5′-ATCAAGAAGGTGGTGAAGCAG-3′ and 5′-TACTCCTTGGAGGCCATGTG-3′.

SYBR Green PCR was performed in optical caps for a 96-well tray (Bio-Rad) using a 25 *μ*l final reaction mixture containing 1 *μ*l of each primer pair (stock 5 *μ*M), 1 *μ*l of the micro-dissected lysed tissue sample, 12.5 *μ*l iQ SYBR Green (Bio-Rad) and sterile water. The reaction mixture was preheated at 95 °C for 5 min, followed by 40 cycles at 95 °C, 57 °C and 72 °C for 30 s each.

All amplification curves generated with SYBR Green from stained tissue showed the typical sigmoid curve. In every run, a negative control was included to exclude false-positive results. Melting curve analysis was implemented to ensure the correct PCR product. Each tissue sample was analysed at least twice for *LASP1* and twice for *GAPDH*.

The relative gene copy number was evaluated on the basis of the threshold cycles (C_T_ values) of the gene of interest C_T_(*LASP1*) and of the internal reference gene C_T_(*GAPDH*).

The C_T_(*GAPDH*)/C_T_(*LASP1*) ratio in benign control breast tissues will reflect non-amplified *LASP1* conditions. In case of a *LASP1* gene amplification in tumour samples, the threshold cycle number will decrease while the values for the C_T_(*GAPDH*)/C_T_(*LASP1*) ratio will increase. The reference range from 10 micro-dissected normal breast tissues was determined as 0.9699±0.0743. Therefore, the expectation interval can be calculated as: *μ*±2 s=(0.821278502↔1.118530727) with *μ* (arithmetic mean) and s (s.d.).

### Cell lines and cell culture conditions

Hepatocellular carcinoma cell lines Hep-3B and Hep-G2, breast cancer cell lines BT-20, MCF-7 and MDA-MB-231, urothelial cancer cell lines T24 and RT-4, glioblastoma cell lines U251MG, U138MG and U87MG, medulloblastoma cell lines DAOY and D283 as well as chorioncarcinoma cell lines JEG-3 and JAR were obtained from Cell Line Services (Heidelberg, Germany) and grown in plastic cell culture flasks in a humidified incubator at 37 °C under 5% CO_2_ atmosphere in RPMI 1640 medium containing 10% heat-inactivated fetal bovine serum (PAA, Linz, Austria) and 1% streptomycin/ampicillin (Invitrogen). Cells were cultured until homogeneous morphology of cells was reached (passage 3–4) because LASP-1 belongs to a group of several differential expressed proteins that are upregulated after later passages (Sun *et al*, 2006).

### p53 Mutations

Cell lines with known p53 mutations are listed in the database: http://p53.free.fr. All mutations result in a non-functional p53 protein: http://p53.iarc.fr.

### Cell cycle synchronisation and FACS analysis

BT-20 cells were rendered quiescent (G_0_) by serum deprivation in RPMI 1640 with 0.1% FCS for 24 h followed by incubation with medium supplemented with 10% FCS to allow cell cycle re-entry in G_1_. To block S-phase transition, cells were incubated in medium supplemented with 10% FCS and 2 *μ*g ml^–1^ aphidicolin for 24 h (Sigma). To synchronise the culture at G_2_/M phase, cells in the S-phase were released in RPMI 1640 medium supplemented with 10% FCS for 12 h.

Cell cycle distribution was monitored by propidium iodide staining and measuring fluorescence in a FACScan 2 (Becton Dickinson, Heidelberg, Germany). BT-20 cells were harvested by trypsination and fixed in 70% ethanol (4 °C) for 1 h followed by incubated in a solution containing 50 *μ*g ml^–1^ RNase in PBS for 30 min. For staining, 50 *μ*g ml^–1^ propidium iodide was added for another 30 min. Cells were analysed by FACS, and the proportion in G_0_/G_1_, S and G_2_/M phases was estimated using Modfit cell cycle analysis programme. Measurements were performed on at least three independent synchronisation experiments.

### Preparation of nuclear and cytosolic cell fractions

Human breast cancer cell lines were harvested at 80% confluence through trypsination. Isolation of nuclei and cytosol was carried out using NE-PER nuclear and cytoplasmic extraction Reagents (Pierce, Bonn, Germany) following the manufacturer's instructions. Samples were solved in Laemmli sample buffer at a final concentration of 1 × 10^6^ ml^–1^ and stored at −20 °C before western blot electrophoresis.

### Western blot analysis

For western blotting, cells were lysed in Laemmli sample buffer. Equal amounts of protein, according to cell count, were resolved by 12% SDS–PAGE. After blotting on nitrocellulose membrane and blocking with 3% non-fat dry milk in 10 mM Tris, pH 7.5, 100 mM NaCl, 0.1% (w/v) Tween 20, the membrane was incubated with the antibody raised against LASP-1 (1 : 20 000) (Butt *et al*, 2003) or PDEF antibody (1 : 1000) followed by incubation with horseradish peroxidase-coupled goat anti-rabbit IgG (Bio-Rad), diluted 1 : 5000, and visualised by ECL (Amersham Biosciences, Freiburg, Germany). Quantification of autoradiography signals was carried out by densitometry using the ImageJ software (NIH, Bethesda, MD, USA).

GAPDH (1 : 1000; Santa Cruz, Heidelberg, Germany) was used as a specific cytosolic marker to exclude cytoplasmic contamination of the nuclei preparation. Anti-Lamin A+C antibody (1 : 50; Abcam, Cambridge, UK) served as a specific nuclear marker to exclude nuclear contamination in cytoplasmic cell samples. At least three independent experiments have been carried out and representative results are shown.

## Results

### Overexpression of LASP-1 in breast cancer is not due to gene amplification

As increasing amounts of contaminating non-malignant cells lead to a significant decrease in detection sensitivity (Kallioniemi *et al*, 1994), we used individual micro-dissected breast cancer cells to examine the *LASP1* copy number in DNA samples from 64 patients with known invasive breast carcinoma selected randomly from January 2000 to December 2007.

We detected only 1 out of 64 tissue samples (1.5%) with a C_T_(*GAPDH*)/C_T_(*LASP1*) ratio higher than the expectation interval, showing a negligible rate of *LASP1* gene amplification. Therefore, the observed overexpression of the LASP-1 protein in more than 55% of human breast cancers (Grunewald *et al*, 2007a) is likely due to reasons distinct from gene amplification.

### LASP-1 overexpression neither correlates with PDEF expression nor with p53 mutations

**Figure 2:**
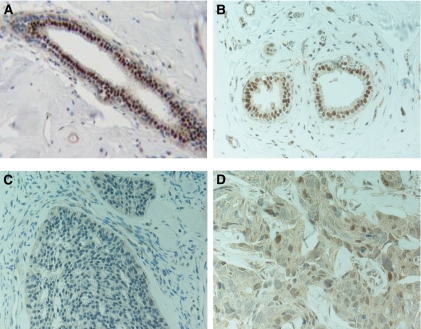
Immunohistochemical staining of PDEF (DAB, brown, magnification × 400) in benign breast tissue (**A** and **B**) and invasive ductal breast cancer samples (**C** and **D**) showing a nuclear PDEF staining in normal tissue and a more cytosolic PDEF localisation in tumour cells.

Commonly, the inner layer of benign ductal luminal epithelial cells show a high nuclear PDEF staining while in invasive ductal carcinoma, a weak PDEF staining is detected mainly in the cytosol (Feldman *et al*, 2003), (Figure 2). To determine whether the reported reciprocal effect of PDEF on LASP-1 in non-invasive and invasive breast cancer cell lines (Turner *et al*, 2008) is transferable to breast tumour patient samples, we evaluated 35 primary breast cancer tissues for PDEF expression; 17 with known high (⩾8) LASP-1 IRS and 18 specimens with low (⩽3) LASP-1-IRS.

In all 35 tested human breast cancer samples, we observed a comparable cytosolic PDEF localisation without significant differences in staining intensity. Only two out of the tested samples showed in parts additional nuclear PDEF staining. No correlation between the PDEF levels in invasive ductal carcinoma and high or low LASP-1 expression could be detected.

**Figure 3:**
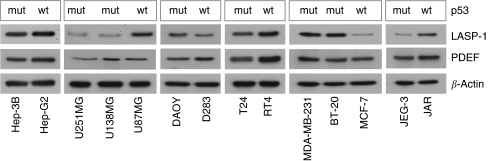
PDEF and LASP-1 protein expression in various cancer cell lines with (mut) and without (wt) known p53 mutations that lead to functionally inactive tumour suppressor protein. *β*-Actin blotting was used as a control for equal protein loading. Cell lines: hepatocellular carcinoma (Hep-G2, Hep-3B), glioblastoma (U251, U13898, U87), medulloblastoma (DAOY, D238), urothelial carcinoma (T24, RT4), breast cancer (MDA-MB-231, BT-20, MCF-7) and chorioncarcinoma (JEG-3, JAR). There is neither a correlation between PDEF and LASP-1 expression nor between LASP-1 protein levels and p53 mutations.

When examining tumour cell lines of different entities, an overall PDEF expression is observed that does not correlate *per se* with low LASP-1 protein concentration (Figure 3). For example, while the PDEF levels are similar in all three tested breast cancer cell lines (MDA-MB-231, BT-20, MCF-7) LASP-1 expression is only reduced in MCF-7 cells. Analogous differences are also observed with glioblastoma and chorioncarcinoma cell lines (Figure 3).

To test whether *LASP1* is transcriptionally regulated by p53, we analysed several human cancer cell lines of different tumour entities with and without known *p53* mutations for LASP-1-expression by western blot. All mutations result in a functionally inactive tumour suppressor. In summary, the analysis showed no correlation between high LASP-1-expression and *p53* mutations (Figure 3). For instance, in spite of the total loss of function because of an additional stop codon in the *p53* gene of the urothelial cell line T24, both, *p53* wild-type RT-4 and *p53* mutant T24 urothelial cancer cell lines express high levels of LASP-1. In contrast, the chorioncarcinoma cell line JEG-3 with a *p53* mutation and the breast carcinoma cell line MCF-7-expressing wild-type p53 show low LASP-1-expression.

### Nuclear localisation of LASP-1 correlates with poor long-term survival

In an earlier case–control study, a strong cytoplasmic staining for LASP-1 was detected in >55% of the invasive tumours, which correlated significantly with increased tumour size and rate of nodal-positivity. In addition, we observed a distinct nuclear LASP-1-localisation pattern that was absent in benign tissue (Grunewald *et al*, 2007a).

We therefore performed a retrospective study (January 1985 until December 2007) with samples of 177 archival cases of confirmed histological diagnosis of invasive breast carcinomas to evaluate the long-term survival of breast tumour patients in relation to nuclear and cytoplasmic LASP-1-positivity.

Cytoplasmic LASP-1 protein expression was detected in 95% of the breast carcinomas; thereof 31% showed an additional nuclear LASP-1 staining (Figure 1, Table 1). A low cytoplasmic expression correlated with negative/low nuclear staining and a high cytoplasmic LASP-1-expression with high nuclear localisation (Table 1).

**Figure 4:**
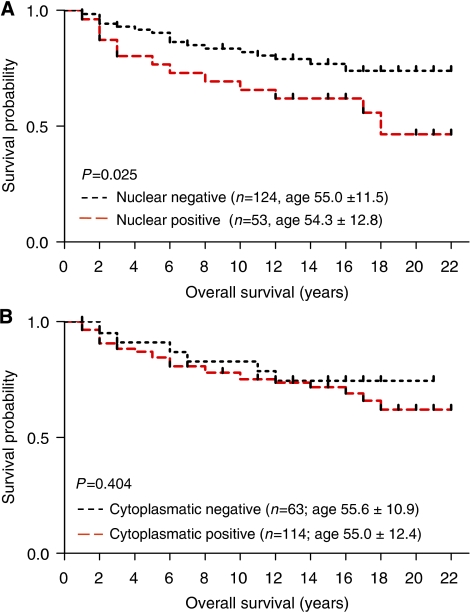
Outcome of patients with nuclear and cytosolic LASP-1 localisation. Kaplan–Meier plots measuring patients OS in years against cumulative survival for nuclear (**A**) and cytosolic (**B**) LASP-1 staining. The analysis included patients diagnosed from 1985 to 2007 (*n*=177). Nuclear LASP-1-positivity is associated with poor OS.

The prognostic effect of nuclear and cytosolic LASP-1 staining was further tested using the Kaplan–Meier survival analysis. There was a significant correlation (*P*=0.025) between patients with positive nuclear LASP-1 staining and poor overall survival (OS) (Figure 4A, Table 2) while there was no significant association between OS and cytoplasmic LASP-1 staining (Figure 4B, *P*=0.404). Surprisingly, a relationship between high positive nuclear staining and low grading (*P*=0.025) was observed, whereas all other clinical parameters analysed (i.e., nodal status, oestrogen and progesterone receptor status, recurrence) did not correlate with nuclear LASP-1-localisation (Table 3).

### Rate of nuclear LASP-1-localisation increases during proliferation and augments in G2/M phase

**Figure 5:**
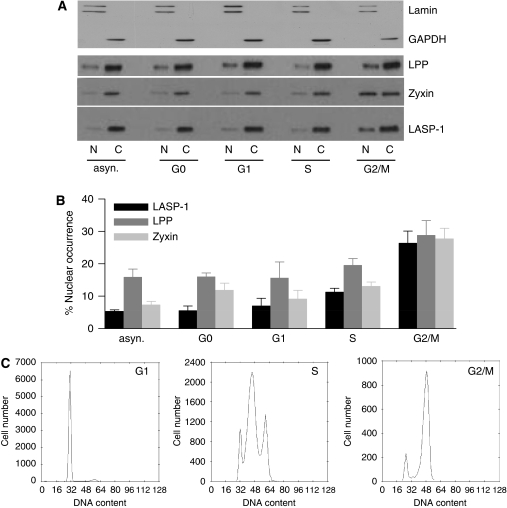
Cell cycle-dependent nuclear and cytosolic LASP-1 distribution. (**A**) Western blotting of nuclear (N) and cytosolic (C) proteins levels of LPP, Zyxin and LASP-1 in BT-20 cells synchronised to G0, G1, S and G2/M phase. Purity of nuclear and cytosolic fractions was controlled by Lamin A+B and GAPDH western blots, respectively. (**B**) Percentage of nuclear LPP, Zyxin and LASP-1 protein concentration (expressed as percentage of total cellular LPP, Zyxin and LASP-1 protein levels, respectively) plotted against cell cycle phases. (**C**) Flow-cytometric analysis of cell cycle phases (propidium iodide staining of total DNA).

There are two facts regarding LASP-1 that seem to be linked: (a) the prominent nuclear localisation of LASP-1 in primary breast cancer (Figure 1) (Grunewald *et al*, 2007a) and (b) cell cycle arrest at G2/M accompanied by reduced cell proliferation after knockdown of LASP-1 in breast carcinoma cell lines (Grunewald *et al*, 2006). Therefore, it was tempting to speculate that the rate of nuclear LASP-1-localisation might be cell cycle dependent. To test this hypothesis, BT-20 cell lysates were subjected to cytoplasmic and nuclear fractioning. Purity of the fractions was confirmed by probing western blot membranes for the nucleus marker Lamin A+B and the cytoplasmic marker GAPDH. Figure 5A shows that there was virtually no cross-contamination.

In cells with an asynchronous cell cycle, in non-proliferating G0 phase cells (resting/senescent) and in G1 phase cells (G1/S-checkpoint), LASP-1 was found primarily within the cytoplasm (95%) (Figures 5A and B). During proliferation (S-phase) the nuclear LASP-1 concentration increased up to 10% and reached a peak at G2/M phase (G2/M) although the overall LASP-1 protein level did not change (data not shown).

As LPP and Zyxin are known binding partners of LASP-1 (Keicher *et al*, 2004; Li *et al*, 2004) and are discussed as possible shuttle proteins to transfer LASP-1 into the nucleus we also controlled their distribution during cell cycle phases. Consistently, like LASP-1, both proteins showed a cell phase-dependent nuclear increase in G2/M (Figure 5B) without changes in absolute protein concentration. All cell cycle phases were controlled by flow cytometry (Figure 5C).

To further validate the influence of nuclear LASP-1 occurence on cell proliferation, we quantified the number of positive stained cells for the proliferation marker Ki67 in 30 breast cancer tissue samples with known high LASP-1 IRS⩾8 and either nuclear or cytosolic LASP-1 localisation.

Although only 30.7% of the invasive ductal carcinoma samples with cytosolic LASP-1 expression are positive for Ki67 staining, 68.7% of the breast cancer tissues with nuclear LASP-1 occurrence show positive staining for the proliferation marker Ki67 (*χ*^2^ test; *P*=0.04).

## Discussion

The *LASP1* gene was initially identified from a cDNA library of metastatic axillary lymph nodes (MLN) from human breast cancer and therefore called *MLN50*. The gene was mapped to chromosomal region 17q11-q21.3, a region known to contain the *c-erbB-2* (HER-2/neu) and the *BRCA1* oncogene and to be altered in 20–30% of all breast cancers (Tomasetto *et al*, 1995a, 1995b). Since its discovery in 1995, several experimental approaches have been carried out to determine the cause of LASP-1 overexpression and its regulatory mechanisms. For instance, LASP-1 overexpression was reported to be due to *LASP1* gene amplification detected in 12 out of 98 tested whole breast cancer samples (Bieche *et al*, 1996) while Tomasetto *et al* detected an amplification of *LASP1* only in one (BT-474) out of eight different breast cancer cell lines (Tomasetto *et al*, 1995b). Others observed deregulation of normal LASP-1-expression in relation to changes in PDEF and urokinase-type plasminogen activator (uPA) concentration or because of loss of p53 tumour suppressor activity (Turner *et al*, 2008; Salvi *et al*, 2009; Wang *et al*, 2009).

However, in this work, we analysed the expression pattern of LASP-1 in primary invasive breast cancers using micro-dissected tissues. Our data clearly show that the *LASP1* gene is not amplified in the vast majority of human breast cancers (only 1 out of 64 cases), suggesting that LASP-1 overexpression is mediated through transcriptional regulation rather than gene amplification. In the context of transcriptional regulation, we revealed that LASP-1 overexpression does not correlate *per se* with defects in the tumour suppressor protein p53 transcriptionally repressing LASP-1 (Wang *et al*, 2009). Although the data for the regulation of *LASP1* gene expression by p53 are convincing, there are clearly additional mechanisms involved in LASP-1 protein upregulation such as transcriptional cofactors and decay rates than just functional defects in p53.

As for PDEF, we could not confirm an association between low PDEF protein expression and high LASP-1 levels although Turner *et al* showed that re-expression of PDEF in cells with low PDEF protein expression resulted in reduced LASP-1 levels (Turner *et al*, 2008).

However, PDEF mRNA concentration and protein expression in breast cancer cell lines is discussed controversially. As reported earlier, PDEF protein detection did not always correspond to PDEF mRNA levels. Although some studies showed increased PDEF mRNA (Turcotte *et al*, 2007) or protein expression in invasive ductal carcinoma (Sood *et al*, 2007) others observed reduced protein expression in breast cancer cells (Feldman *et al*, 2003; Doane *et al*, 2006; Ghadersohi *et al*, 2007; Turner *et al*, 2008). Recently, this discrepancy was explained by the identification of two microRNAs in human breast tumour samples that directly repressed PDEF protein expression in spite of the detection of high PDEF mRNA concentration (Findlay *et al*, 2008).

In a recent paper by Grunewald *et al*, LASP-1 was reported to be highly expressed in invasive breast carcinomas compared with fibroadenomas. Strong cytoplasmic staining for LASP-1 was found in 55.4% of the invasive breast tumours (Grunewald *et al*, 2007a). In addition to the reported localisation at focal contacts and lamellipodia, a perinuclear and nuclear distribution of the protein was observed. These data hint to a potential additional signalling function of LASP-1 as a shuttle protein thereby transducing growth signals from the sites of cellular contacts with the ECM into the nucleus.

In support of this hypothesis, this work shows a cell cycle-dependent increase of nuclear LASP-1 during the mitotic G2/M phase in proliferating tumour cells (Figure 5C) while serum-starved quiescent cells (G0) as well as cells in G1 and S-phase show only minor levels of the protein in the nucleus. Our observations are consistent with earlier data showing a specific cell cycle arrest at G2/M and inhibition of cell proliferation after LASP-1 knockdown in breast and ovarian cancer cell lines (Grunewald *et al*, 2006, 2007a, 2007b). In reverse, a high LASP-1 concentration in the nucleus would show sustained cell proliferation. In fact, we found that approximately 70% of the patient samples with nuclear LASP-1 staining were positive for the cell proliferation marker Ki67 while only 30% of the patients with cytosolic LASP-1 expression showed positive Ki67 staining.

Consistently, earlier studies revealed a correlation between LASP-1-expression and tumour size as well as nodal-positivity in human breast carcinoma (Grunewald *et al*, 2007a). The present continuative long-term follow-up strengthens the assumed link between increased nuclear LASP-1-localisation and poor survival of patients with breast cancer suggesting an effect of nuclear LASP-1 on cell proliferation, especially because the absolute amount of cytosolic LASP-1-expression does not correlate with patients' OS.

Unexpectedly, we found a high nuclear localisation of LASP-1 in differentiated G1 tumours while in parallel nuclear LASP-1 abundance was correlated with worse prognosis. It is possible that tumours with a high nuclear LASP-1-expression represent a subgroup with poor survival irrespective of the grading. This could, for example, be due to a decreased response to endocrine or chemotherapeutic treatment. However, the number of available G1 tumours was very low. Therefore, we will not draw definitive conclusions regarding these data.

On the molecular level, the zinc-finger containing LIM-domain of LASP-1 offers a possibility for direct binding to DNA (Hammarstrom *et al*, 1996). LASP-1 may even form heterodomains to become a nuclear transcription factor (Kadrmas and Beckerle, 2004).

Although LASP-1 sequence analysis revealed no nuclear localisation signal, the classical import pathway for the nucleus (Kutay and Guttinger, 2005), LASP-1 binds to the well-characterised shuttle proteins and transcription factors LPP and Zyxin that are upregulated in a wide variety of human cancers (Beckerle, 1997; Petit *et al*, 2003; Keicher *et al*, 2004; Li *et al*, 2004; Grunewald *et al*, 2009). For Zyxin, it is known that during mitosis a fraction of the cytoplasmic-dispersed protein becomes phosphorylated (most likely by Cdc2 kinase) and associates with the tumour suppressor h-warts (LATS1), a key governor of G2/M-progression, at the mitotic apparatus (Hirota *et al*, 2000).

Our data suggest that pathophysiological localisation of LASP-1 in the nucleus of malignant cells may induce mitosis and thereby enhance cell proliferation, possibly in concert with Zyxin and LPP. Further work will be needed to identify the nuclear shuttle partner(s) of LASP-1, the mechanism of nuclear translocation and the regulation of cell cycle progression.

The present continuative long-term follow-up provides evidence for the relation of increased nuclear LASP-1-localisation and poor survival of patients leading to the question whether nuclear LASP-1-positivity defines a subgroup of patients with unfavourable prognosis that is not responding to conventional treatment approaches. Future work is on the way to elucidate the precise molecular and clinical effect of LASP-1 nuclear overexpression.

## Figures and Tables

**Table 1:** Analysis of overall survival in relation to LASP-1 staining intensity

**LASP-1 staining intensity**	**No. of patients**	**Positive nuclear LASP-1 staining**	**No. of deceased patients**	**Overall survival (%)**
Score 0	9	0 (0%)	0	100
Score 1	46	6 (13%)	7	84.8
Score 2	109	46 (42.2%)	27	75.2
Score 3	13	4 (28.5%)	4	69.2
Total	177	56 (31%)	38	89.6

Abbreviation: LASP-1=LIM and SH3 protein 1.

**Table 2:** Analysis of overall survival in relation to nuclear LASP-1-positivity

**Nuclear LASP-1-positivity**	**No. of patients**	**No. of deceased patients**	**Overall survival (%)**
No	121	21	82.6
Yes	56	17	69.6

Abbreviation: LASP-1=LIM and SH3 protein 1.

**Table 3:** Univariate analysis of positive nuclear LASP-1 staining and clinicopathological parameters

**Parameters**	**Positive nuclear LASP-1 staining**	***P*-value**
*Nodal status*		
N+ (*n*=83)	31 (37.3%)	
N− (*n*=94)	25 (26.6%)	0.2 (F)
		
*Tumour size*		
Tis (*n*=3)	0 (0.0%)	
T1 (*n*=108)	37 (34.3%)	
T2 (*n*=51)	15 (29.4%)	
T3 (*n*=5)	2 (40.0%)	
T4 (*n*=10)	2 (20.0%)	0.69 (M)
		
*Metastasis*		
M+ (*n*=6)	1 (16.7%)	
M− (*n*=171)	55 (32.2%)	0.67 (F)
		
*Grading*		
G1 (*n*=7)	4 (57.1%)	
G2 (*n*=69)	27 (39.1%	
G3 (*n*=45)	9 (20.0%)	0.03 (M)
		
*Recurrence*		
Yes (*n*=36)	8 (22.2%)	
No (*n*=87)	27 (31.0%)	0.38 (F)
		
*ER*		
ER+ (*n*=98)	28 (28,6%)	
ER− (*n*=62)	23 (37.1%)	0.3 (F)
		
*Progesteron receptor*		
PR+ (*n*=86)	22 (25.6%)	
PR− (*n*=71)	27 (38.0%)	0.13 (F)
		
*HER2/neu*		
Her+ (*n*=27)	6 (33.3%)	
Her− (*n*=47)	18 (28.1%)	0.77 (F)

Abbreviations: ER=oestrogen receptor; F=Fisher's exact test; LASP-1=LIM and SH3 protein 1; M=Mann–Whitney Test; PR=progesterone receptor.

Statistical significance is assumed when *P*<0.05.
