# Supplementary material for: Nuclear localisation of LASP-1 correlates with poor long-term survival in female breast cancer
Source: Br J Cancer. 2010 May 11;102(11):1645–53. doi: 10.1038/sj.bjc.6605685 (PMC2883150; doi:10.1038/sj.bjc.6605685)
Supplement: Supplementary Figures 1 and 2 [file 6605685x1.ppt]

## Slide 1
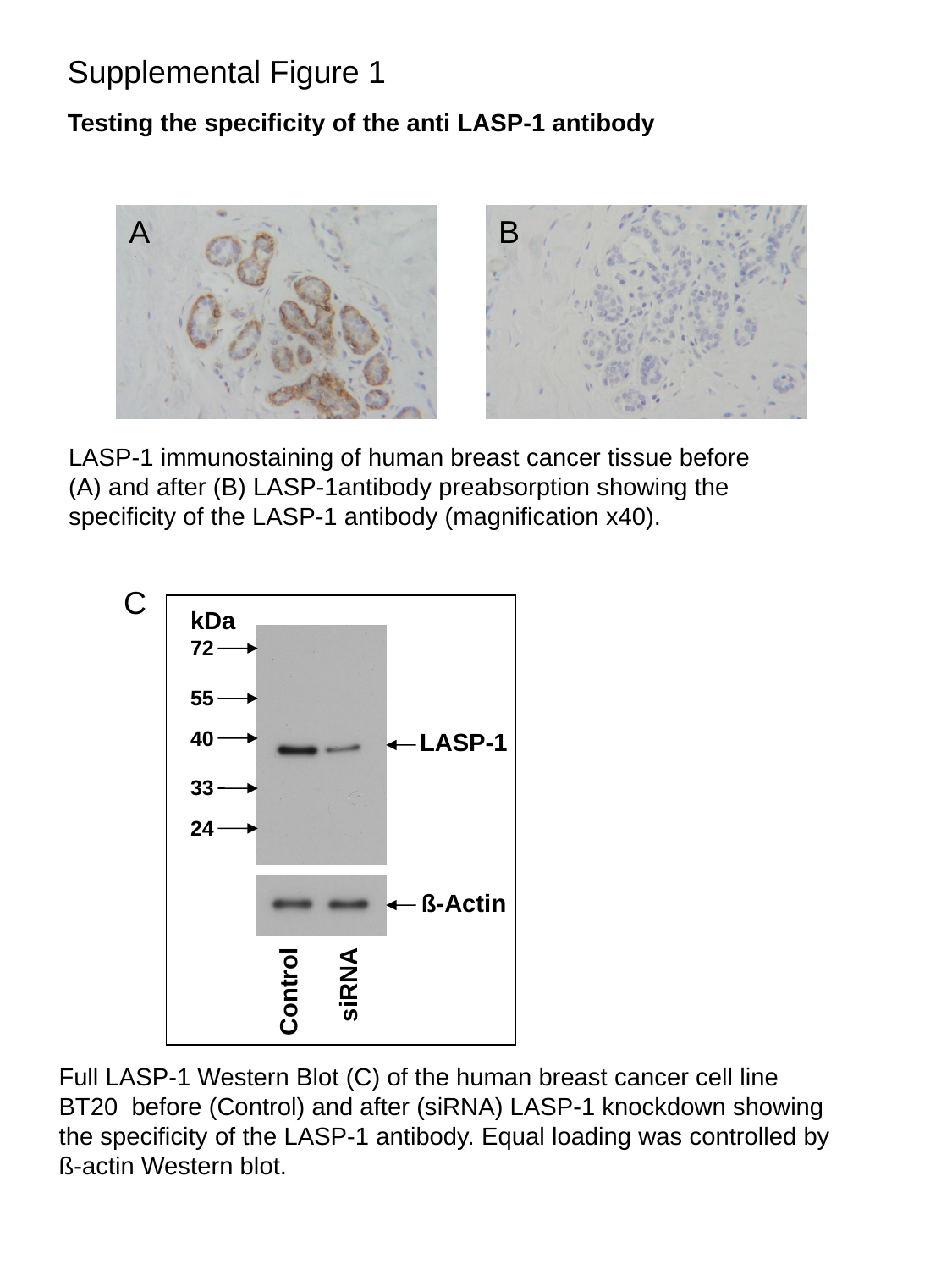

Supplemental Figure 1
Testing the specificity of the anti LASP-1 antibody
A
B
LASP-1 immunostaining of human breast cancer tissue before
(A) and after (B) LASP-1antibody preabsorption showing the
specificity of the LASP-1 antibody (magnification x40).
C
kDa
72
55
40
LASP-1
33
24
ß-Actin
siRNA
Control
Full LASP-1 Western Blot (C) of the human breast cancer cell line
BT20 before (Control) and after (siRNA) LASP-1 knockdown showing
the specificity of the LASP-1 antibody. Equal loading was controlled by
ß-actin Western blot.

## Slide 2
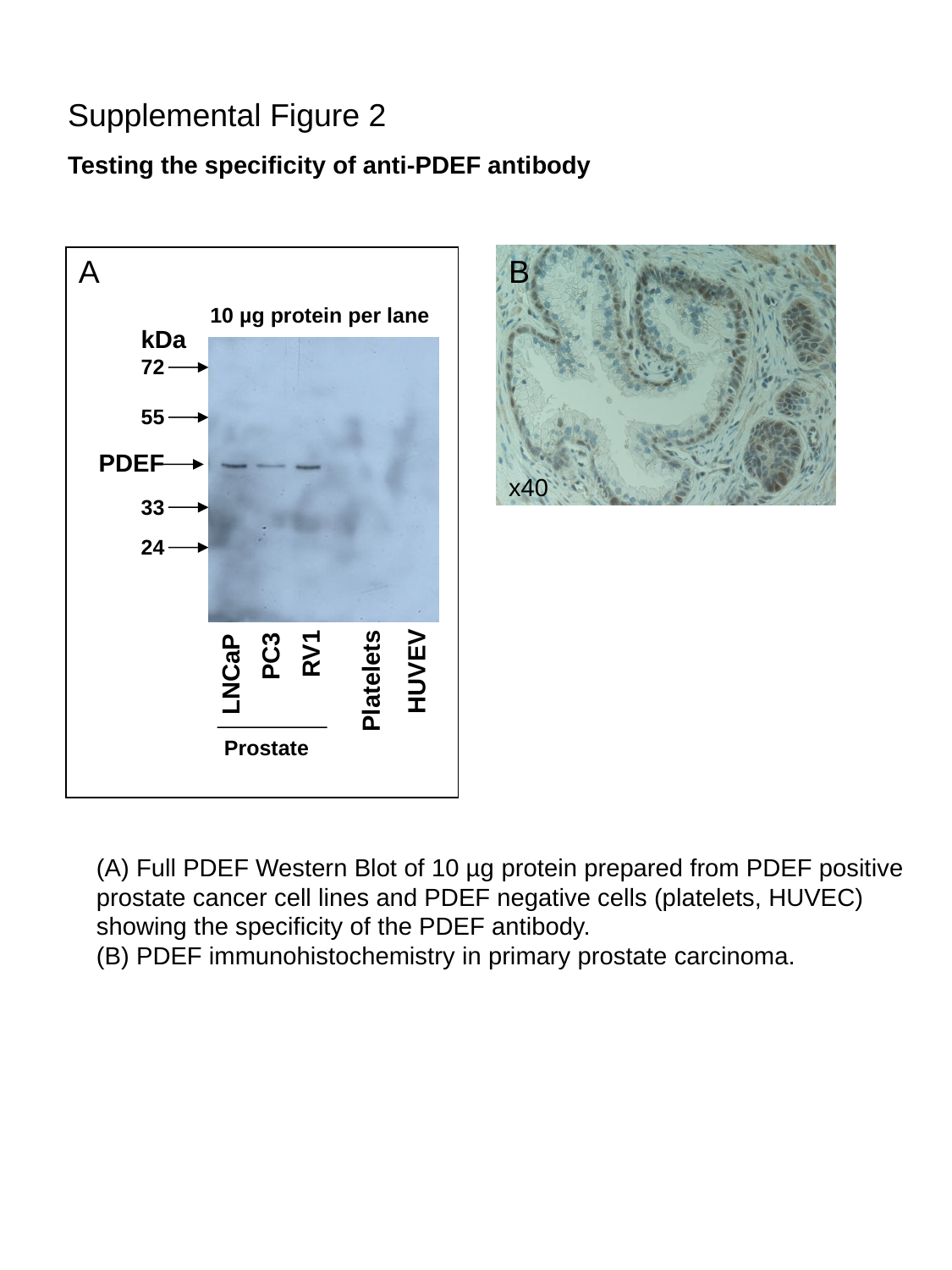

Supplemental Figure 2
Testing the specificity of anti-PDEF antibody
A
B
10 µg protein per lane
kDa
72
55
PDEF
33
24
RV1
PC3
HUVEV
LNCaP
Platelets
Prostate
x40
(A) Full PDEF Western Blot of 10 µg protein prepared from PDEF positive
prostate cancer cell lines and PDEF negative cells (platelets, HUVEC)
showing the specificity of the PDEF antibody.
(B) PDEF immunohistochemistry in primary prostate carcinoma.
